# PDZ domains and their binding partners: structure, specificity, and modification

**DOI:** 10.1186/1478-811X-8-8

**Published:** 2010-05-28

**Authors:** Ho-Jin Lee, Jie J Zheng

**Affiliations:** 1 Department of Structural Biology, St. Jude Children's Research Hospital, Memphis, TN 38105, USA

## Abstract

PDZ domains are abundant protein interaction modules that often recognize short amino acid motifs at the C-termini of target proteins. They regulate multiple biological processes such as transport, ion channel signaling, and other signal transduction systems. This review discusses the structural characterization of PDZ domains and the use of recently emerging technologies such as proteomic arrays and peptide libraries to study the binding properties of PDZ-mediated interactions. Regulatory mechanisms responsible for PDZ-mediated interactions, such as phosphorylation in the PDZ ligands or PDZ domains, are also discussed. A better understanding of PDZ protein-protein interaction networks and regulatory mechanisms will improve our knowledge of many cellular and biological processes.

## Introduction

Diverse biological activities are regulated through the dynamic interactions of modular protein domains (e.g., WW, SH3, SH2, PH, and PDZ) and their corresponding binding partners [1]. Elucidation of the specificity, selectivity, and regulatory mechanisms involved in these protein-protein interactions can therefore provide important insights into biological processes such as cell proliferation and cell polarity [1,2].

**Figure 1:**
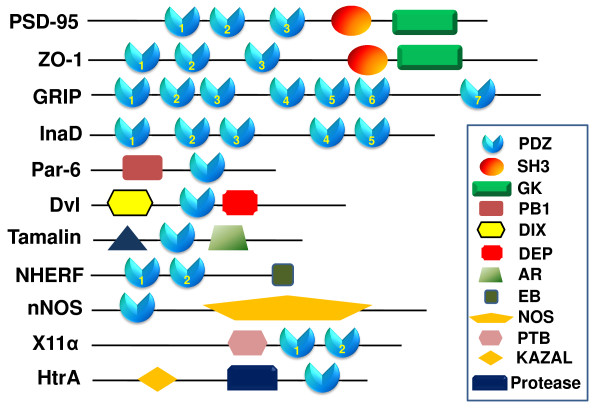
**Examples of PDZ domain-containing proteins. **Proteins are indicated by black lines scaled to the length of the primary sequence of the protein (from SMART)[184].

PDZ domains are abundant protein-protein interaction modules found in various species (Figure 1) [3-6]. In the mouse genome, for example, 928 PDZ domains have been recognized in 328 proteins, which exist in single or multiple copies or in combination with other interaction modules (Figure 1) [7]. From the abundance and diversity of PDZ domains in cells it is apparent that many cellular and biological functions, especially those involving signal transduction complexes, are affected by PDZ-mediated interactions [7-20].

PDZ domains are small and often modular entities consisting of 5 or 6 β-stranded and 2 or 3 α-helical structures [21]. PDZ domains typically recognize the extreme C-termini of target proteins [22], but some also recognize the internal sequence motif of target proteins through a single binding site on the domains [23-25]. Structural analysis of PDZ domains and PDZ-mediated interactions by NMR and X-ray crystallographic methods in conjunction with computational methods has provided insights into the specificity or promiscuity of PDZ protein-protein interactions [26,27]. Proteomic methods, such as large scale protein arrays [28-30] and peptide libraries [31-44], have also been used to understand the binding properties of PDZ protein-protein interactions at a genome-wide level, which may provide clues about novel functions of proteins of interest in various cells. PDZ-containing proteins interact with many proteins within cells, so studying the regulatory mechanisms of PDZ protein-protein interactions, such as phosphorylation, autoinhibition, and allostery, is also vital to understand their biology. This review focuses on the advances made in the fields of structural biology, proteomic applications, and regulatory mechanisms of PDZ-mediated interactions.

## Structural characteristics of PDZ domains

At present, more than 200 structures of PDZ domains - either the PDZ domains alone, their complexes with binding partners, or PDZ-PDZ dimers - have been determined by NMR and X-ray crystallography [26]. Small-angle X-ray scattering (SAXS) in combination with NMR has also been used to determine the structure of PDZ-containing proteins [45]. These structural studies provide detailed information on ligand recognition and selectivity of PDZ-containing proteins at the molecular level. In this section, we discuss the recent advances in understanding the structural characteristics of isolated PDZ domains, and PDZ domains in complexes with their binding partners.

### PDZ domains structures

#### Canonical PDZ domains

**Figure 2:**
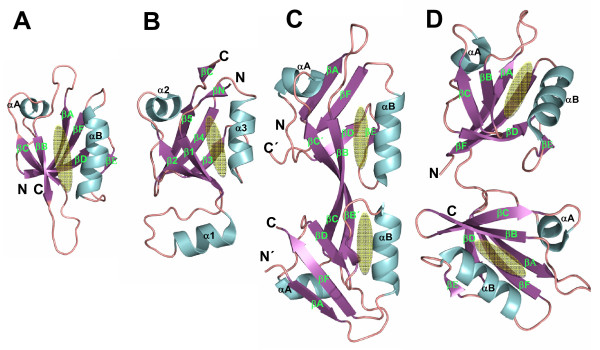
**Structures of PDZ, PDZ-like, PDZ-PDZ dimer, and tandem PDZ domains**. (A) Ribbon diagram of Dvl-1 PDZ (PDB code: 2KAW). (B) HtrA2 PDZ (PDB code: 1LCY). (C) ZO-1 PDZ2 (PDB code:2RCZ). (D) GRIP-1 PDZ1+2 (PDB code: 2QT5). The binding site of each PDZ domain is shown by a yellow oval. The structures were generated using the Pymol software.

PDZ domains are usually 80-100 amino acid residues long and adopt a similar topology: structural studies have revealed that canonical PDZ domains are usually composed of 6 β-strands (βA ~ βF), a short α-helix (αA) and a long α-helix (αB) (Figure 2A) [21,46,47]. The canonical PDZ family has a highly conserved fold, but secondary structures vary in length [5,20,47-49]. The N- and C-termini of canonical PDZ domains are in proximity to each other on the opposite side from the peptide-binding site in a groove between the αB-helix and βB-strand structures (Figure 2A).

#### PDZ-like domains

Similar to canonical PDZ domains, the HtrA family, including HtrA (or DegP), DegS, and DegQ, adopt a PDZ-like fold consisting of 5 β-strands (β1-β5) capped by 2 α-helices (α2 and α3) and also 2 short β-strands at the N and C termini (βN and βC). The well-defined α-helix (α1) is formed in the region between the β1 and β2 loop of the PDZ-like domain (Figure 2B) [17,41].

### Dimerization of PDZ domain

Although most PDZ domains that have been studied in isolation are found to be monomers [5,20,47-49], some form dimers [50-55]. Shank-1 PDZ and GRIP-1 PDZ6, for example, form a homodimer via the conserved βB/βC loop and N-terminal βA strands, which display an antiparallel orientation between the βA strands of the proteins [50,51]. The formation of the PDZ dimer does not affect the binding to its partner because the peptide-binding sites of both PDZ domains remain open. However, the role of both PDZ dimerizations found *in vitro *remains unclear, because there is no evidence that the full length Shank1 and GRIP1 proteins form functional dimers *in vivo*.

A novel dimerization mode of PDZ domains has recently been reported in NMR and X-ray crystallographic studies [52-55]. Two independent groups have shown that the second PDZ domain (PDZ2) of the ZO protein forms a dimer by extensive symmetrical domain swapping of β-strands (Figure 2C) [52-55]. The ZO-1-PDZ2 dimer largely maintains the canonical PDZ fold; however, the N- and C-termini of ZO-1-PDZ2 are not close to each other [52-55]. In the ZO-1-PDZ2 dimer, the binding site opens to allow interaction with the target protein [52-54]. *In vivo*, the presence of the ZO-1 dimer or higher order oligomers was confirmed by the co-immunoprecipitation of fluorescent-labeled ZO-1 (GFP-ZO-1) and endogenous ZO-1 [52].

### Tandem PDZ domains

Although isolated PDZ domains usually fold into a well-defined native structure, recent studies have shown that some PDZ domains need other PDZ domains that are connected to them in a tandem arrangement in order to fold properly [56,57]. For example, the first and second PDZ domains (PDZ12) of GRIP-1 proteins are connected in this way (Figure 1). Structural studies have revealed that the isolated PDZ1 of GRIP-1 is not well folded, but that the PDZ12 tandem is (Figure 2D), indicating that the folding of PDZ1 strictly depends on the covalent attachment of PDZ2 [56]. Another example of a tandem PDZ domain is the fourth and fifth PDZ domains (PDZ45 tandem) of GRIP-1, which are required to interact with GluR2/3 [57]. The solution structure of the PDZ45 tandem shows that PDZ4 contains a deformed binding groove, but that this deformed groove stabilizes the structure of PDZ5.

Besides these examples, recent studies reported the structures of tandem PDZ domains present in PSD-95 [58], human synthenin [59,60], and X11a [61], showing that tandem PDZ domains play crucial roles in the formation of structural and functional supramodules [56-63].

### The carboxylate-binding site of PDZ domains

**Figure 3:**
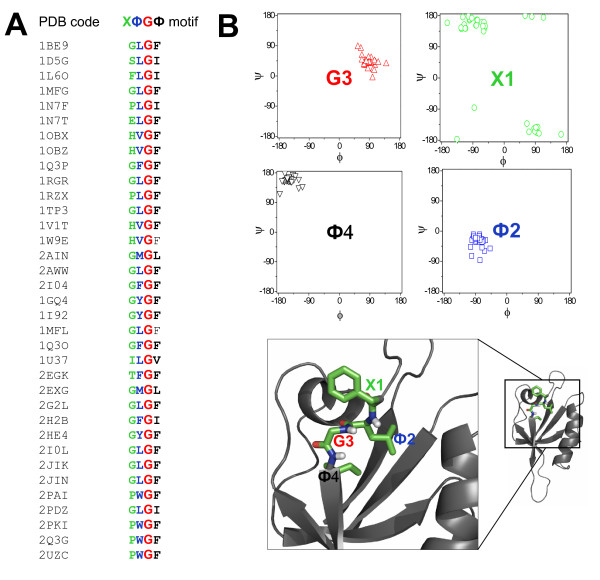
**The amino acid residues in the carboxylate-binding loop regions of PDZ domains adopt a specific conformation**. (A) Selected 'X-Φ-G-Φ' motifs from known structures of PDZ domains, where X represents any amino acid residue and Φ represents hydrophobic amino acid residues. (B) 4 Top boxes: Distribution of the (ϕ,ψ) angle of the amino acid residue at the specific position. Bottom: Ribbon diagram of the Dvl1 PDZ domain (grey, PDB code: 2KAW); the X1-Φ2-G3-Φ4 motif is highlighted (stick).

PDZ domains have a single binding site in a groove between the αB and βB structural elements with a highly conserved carboxylate-binding loop (R/K-XXX-G-Φ-G-Φ motif, where X is any amino acid residue and Φ is hydrophobic residues) located before the βB strand [4,31]. The first Gly residue in this motif is variable among canonical PDZ domains, and can be replaced by a Ser, Thr, or Phe residue (Figure 3A) [26]. The second and the fourth residues are hydrophobic, such as Val, Ile, Leu, or Phe (Figure 3A). The side chains of both of these residues create the hydrophobic binding pocket of canonical PDZ domains [5].

Because this loop region of PDZ domain plays a key role in ligand binding, the conformational properties of amino acid residues in this region of multiple currently deposited PDZ domain structures were analyzed. Interestingly, the second residue in the loop region adopts the α-helix conformation whereas the fourth residue adopts the β-sheet conformation (Figure 3B). The carboxyl oxygen atom of the second residue in the loop region forms a hydrogen bond with a residue in the αA helix, thereby stabilizing this short helix. The third Gly residue in the loop region is fully conserved (Figure 3A) and adopts the left-handed α-helical conformation in structures available to date (Figure 3B), which may be important for determining the PDZ fold. These specific conformations for each residue in the carboxylate-binding loop region allow amide groups to serve as the H-bonding donors (Figure 3B).

As seen in canonical PDZ domains, PDZ-like domains also have a single binding site located in the groove between the β1 strand and the α3 helix structures. The carboxylate-binding loop region [X1-Φ2-G3-Φ4 motif] in PDZ-like domains is located before the β1 strand (Figure 2B), and the third Gly residue is highly conserved in this loop region. The second and fourth residues in the loop regions are hydrophobic residues, as seen in canonical PDZ domains.

## Conformational properties of peptides bound to PDZ domains

### C-terminal regions of proteins interacting with PDZ domains

**Figure 4:**
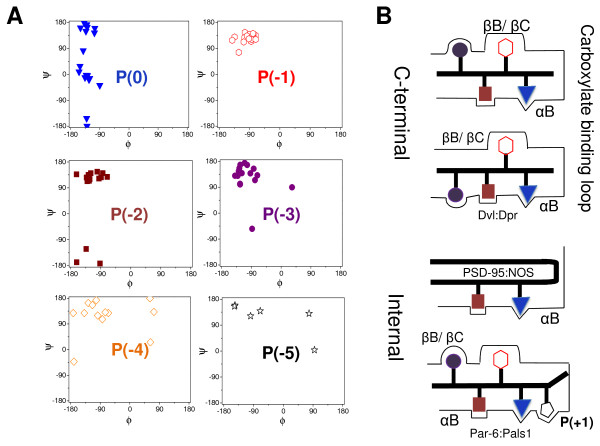
**Each residue in the PDZ-bound ligand adopts a specific conformation**. (A) Distribution of (ϕ,ψ) angles of each residue of the PDZ-bound ligand. Dataset were from PDBsum http://www.ebi.ac.uk/pdbsum/. (B) The orientation of each residue in the side chain of the PDZ ligand in case of a free carboxy-terminal peptide and the internal sequence from PSD-95/nNOS and Par-6/Pals1 complex [65,69,185,186].

To date, approximately 20 structures of PDZ domains in complex with their binding peptides, mostly complexes with free COOH-terminal peptides, have been reported [26]. The binding peptide forms an additional β strand in the groove between the βB strand and the αB-helix structure of the PDZ domain [4]. As the conformational properties of the bound PDZ ligands might be useful to understand, the binding specificity of PDZ domains, the backbone (ϕ,ψ) dihedral angles for each position of the amino acid residues were investigated (Figure 4A). Bound peptides that form complex structures with PDZ domains have 4 or 9 residues. The (ϕ,ψ) angles at the p(0) site of the PDZ ligand (i.e. the most C-terminal residue) are randomly scattered in the Ramachandran plot and do not show preference for a specific conformation. The (ϕ,ψ) angles at p(-1), p(-2), and p(-3) sites are located on the β strand (β_S_) or extended (ε) conformation regions [64]. Interestingly, for the complex structure of the PDZ domain of Dishevelled with the Dapper peptide, the p(-3) residue adopts the right α-helix (α_R_) conformation, which contrasts what is seen in other PDZ-mediated interactions (see also Figure 4B) [65]. Positions -4 and -5 of amino acid residues adopt the β-strand or extended conformation, but some positions have different conformations. Taken together, the difference in conformational properties of each residue at the different positions may explain the binding specificity of PDZ domains (Figure 4A).

### Internal sequence of target proteins

Although the binding of many PDZ-containing proteins occurs by recognition of the extreme C-terminus of target proteins [31], some PDZ domains can also bind to internal sequences of target proteins [48,49,66-70]. The most well-characterized example of this nature is the interaction between the PDZ domain of the syntrophin (or PSD-95) protein with the internal β-hairpin finger structure of the nNOS protein (Figure 4B) [66-68]. This nNOS β-hairpin pockets in the binding site of syntrophin (or PSD-95) protein, which mimics a normal peptide ligand, but the extreme C-terminus is replaced with a sharp β-turn [68]. Another example is the interaction of the internal ligand of the Pals1 protein with the Par-6 PDZ domain (Figure 4B) [69]. Prehoda and coworkers determined the three-dimensional structure of the Par-6 PDZ domain in complex with the Sdt/Pals1-derived peptide Ac-Y^-8^P^-7^K^-6^H^-5^R^-4^E^-3^M^-2^A^-1^V^0^**D^+^**^1^C^+2^P^+3^CONH_2 _by X-ray crystallography. The Pals1 internal ligand adopts the extended conformation, which is comparable to that seen in the nNOS-PSD-95 internal interaction [66,69]. The binding of the Pals1 internal ligand induces a conformational change in the carboxylate-binding loop of the PDZ domain of Par-6, which may result from the formation of salt bridges between the Asp(+1) residue from the internal ligand and a Lys residue from the carboxylate-binding loop, as indicated by alanine scanning mutagenesis experiments [69]. Besides these 2 interactions with internal peptides, several others have also been reported: binding of the PDZ of Dvl with the internal KTxxx(W/I) motif of Frizzled and Idax proteins [48,70], the PDZ binding of nNOS to the internal -[D/E]-x-F-[D/E]- motif of Vac14, and the PDZ interaction of HtrA1/2/3 with internal sequences of misfolded polypeptides [41]. Whether the internal sequences of target proteins adopt a specific conformation in the bound state remains to be determined.

## Interactions among residues in the PDZ - peptide complex

As the C-terminal region of PDZ-binding proteins forms an additional β-strand in the groove between the βB-strand and the αB-helix structure of the PDZ domain [4], each residue in the PDZ ligand can interact with specific residues in the binding pocket of the PDZ domain (Figure 4B). This section summarizes the structural characteristics of these specific interactions among the side chains of PDZ ligands and the binding surfaces of PDZ domains (Figure 4B).

Structural analyses have shown that the p(0) side chain of the PDZ ligand interacts with βB-1, αB-8, and αB-5 side chains of the PDZ domain [32,36,37,41,42,71-73]. The numbers used here in combination with the structural elements represent the position of the relevant amino acid residue on a specific secondary structure element: for example, βB-1 is the first residue of the βB structure. The preference of the p(0) residue is likely related to the size of the βB-1 side chain [36]. If βB-1 is a Phe residue, the p(0) site of the PDZ ligand prefers a Val residue over a bulky residue; on the other hand, if βB-1 is a Leu/Ile residue, the p(0) site of the PDZ ligand prefers bulky residues [73].

The p(-1) side chain of the PDZ ligand may interact with the βB-2 and βC-5 residues or a residue of the βC-αA loop regions, or both, within the PDZ domain. As the p(-1) residue of the PDZ ligand is exposed to the solvent, the residue was initially thought to have no preference. Accumulating evidence, however, shows that some PDZ domains favor specific residues at the p(-1) position [36,41,42,73-75]. For example, the Erbin and Dishevelled PDZ domains prefer a Trp residue at p(-1) [36,46]. To understand why the Trp(-1) residue is preferred in the binding of Dvl-1 PDZ to the VWV tripeptide, its complex structure was determined by NMR spectroscopy, followed by molecular dynamic simulation and assessment of the molecular mechanics with the Poisson-Boltzmann surface area method [46]. The results showed that hydrophobic interactions contribute to the increased binding affinity of the Dvl PDZ/the VWV tripeptide [46]. For the preferred Trp of the p(-1) site for the Erbin PDZ ligand, Beuming *et al*. (2009) predicted a favorable release of high-energy water molecules into bulk [76]. Despite the preference for the W(-1) residue in some PDZ ligands, PDZ domains with Cys residue at βB-2 position likely favor the Cys residue at the p(-1) site in the PDZ ligand [77]. For example, the N-terminal PDZ domain of InaD forms the complex with the C-terminus of NorpA through disulfide bond formation between the Cys residue at p(-1) site in PDZ-ligand and the Cys residue at βB-2 position of the PDZ domain [77]. Kimple *et al*. (2001) proposed that some PDZ domains may also form an intermolecular disulfide bond between a PDZ domain and its binding ligand [77].

The p(-2) residue in the PDZ ligand can interact with αB-1 and αB-5 residues on the PDZ domain, which plays an important to role in determining the binding specificity of PDZ-mediated interactions [4,31,73]. The preference for the p(-2) residue is likely related to the physicochemical properties of αB-1 and αB-5 residues. It has been suggested, for example, that the preference for the Ser or Thr residue at the p(-2) site in the PDZ ligand is due to hydrogen bond formation with the side chain of the His residue at αB-1 [78]. The hydrophobic properties of αB-5 residue may explain the preference of the Thr residue over the Ser residue at the p(-2) site in the PDZ ligand [39,79].

For the p(-3) residue in PDZ ligands, it appears to be difficult to define strict parameters for the interaction. It can interact with the βB-4 for short ligand side chains or the βB-5 residue for long ligand side chains [36,41,80]. However, the p(-3) residue of the PDZ ligand, dapper, is in proximity to the αB-1 residue (Asn) on the Dvl PDZ domain (Figure 4B) [65].

## Characterization of PDZ-mediated interactions with advanced tools

While the complex structures of PDZ domains and their ligands by NMR and X-ray provide molecular details of PDZ-mediated interactions, advanced tools such as proteomics and protein arrays have been developed to characterize the PDZ-mediated interaction network proteome-wide. This section summarizes techniques such as yeast two-hybrid (Y2H), coimmunoprecipitation, protein microarray, and peptide libraries and their applications in studying the PDZ-mediated interactions [79,81-87]. We summarize the classification of PDZ domains investigated by peptide library approaches and suggest a need to deposit the accumulated information obtained by these advanced tools into publicly available databases to accelerate the identification of novel PDZ-mediated interactions.

### Techniques for studying the PDZ-mediated interactions

#### Y2H approach

The Y2H approach is widely used to identify protein-protein interactions [79,81-86]. In a study of PDZ-mediated binding events by Lee and coworkers, the C-terminal fragment of target proteins was subcloned into a bait vector containing a DNA-binding domain, and the PDZ domains subcloned into the matching prey vector containing the corresponding activation domain [84]. Both partial fusion proteins were expressed in the same yeast cell and their binding reconstituted a functional transcription activator, which led to transcriptional activation of a reporter gene. Gisler *et al*. (2008) developed a modified membrane yeast two-hybrid (MYTH) system to test interactions between full-length integral membrane proteins and their cognate PDZ-interacting partners [85]. However, Y2H approaches have a high rate of false positives and false negatives, and therefore their results need to be interpreted with caution [82,83].

#### Co-immunoprecipitation (co-IP) approach

In co-IP, it is attempted to identify a specific protein as interaction partner of another in cellular or tissue protein extracts [84]. The protein complex is immobilized via a specific antibody on protein A or protein G Sepharose beads and unbound proteins are removed by a series of washes. The protein complex is then eluted from the beads and analyzed by SDS-PAGE followed by Western blotting with specific antibodies for the bait and prey [84,88]. Co-IP is usually used as a complementary assay to confirm the direct interaction identified by other biophysical methods [81,84,86,89-92]. Gee *et al*. (2009), for example, identified a direct interaction of the C-terminus of the vasoactive intestinal polypeptide (VIP) type -1 receptor (VPAC1) and the PDZ domain of the synaptic scaffolding molecule (S-SCAM) by an Y2H screen, which was then confirmed by co-IP in HEK293 mammalian cells and human pancreatic and colonic tissues [92].

#### PDZ domain arrays

PDZ domain arrays have been developed by several groups [28-30]. Hall and coworkers developed a proteomic array of 96 putative class I PDZ domains derived from cytoplasmic proteins [28]. These PDZ domains were expressed as His- and S-tagged fusion proteins [93], purified, and spotted onto 96-grid nylon membranes [28]. Tymianski and coworkers cloned all publicly known human PDZ domains (more than 160 constructs) into expression vectors and produced GST-PDZ fusion proteins, which were arrayed into 96-well plates [94].

Because approximately 20% of GPCR proteins (including ionic channels, ionotropic receptors, and single transmembrane proteins) in the human genome have a PDZ-binding motif, PDZ domain arrays have been repeatedly used to identify the binding partners of GPCR proteins [28-30]. For example, Hall and coworkers demonstrated in this way that the C-termini of GPCR proteins such as P2Y1R, mGluR5, and β1AR are binding partners for several PDZ domains [28-30]. These PDZ protein-protein interactions were subsequently confirmed by coimmunoprecipitation and immunofluorescence co-localization studies [28-30].

PDZ domain arrays also have diagnostic applications and can be used to develop biomarkers for detecting viral infections. For example, in a large-scale sequencing analysis of avian influenza viruses (AIVs), Naeve and coworkers found that the multifunctional NS1 protein has a PDZ-binding motif at the last C-terminal sequence [95]. By using commercial PDZ domain arrays containing 123 PDZ domains derived from the human genome, they showed that the full-length avian NS1 protein binds to 30 different human PDZ domains, whereas the human NS1 protein binds very weakly or not at all [95]. These results indicate that avian NS1 proteins, not human NS1 proteins, bind to and inhibit many PDZ interactions. Lamb and coworkers verified the importance of these PDZ-mediated interactions in a mouse model [96]. A company has recently developed a rapid antigen assay test to detect the avian influenza virus in humans using its own PDZ array http://www.cdc.gov/EID/content/14/3/ICEID2008.pdf.

Although most PDZ domain arrays developed to date provide qualitative information on PDZ protein-protein interactions, PDZ domain arrays and fluorescence polarization can be combined to generate quantitative information of PDZ-mediated interactions. This information can be used to further elucidate the specific binding properties of PDZ domains [97]. For example, MacBeath and coworkers generated microarrays containing 157 mouse PDZ domains [97]. These PDZ domains were arrayed onto aldehyde-presenting flat glass slides, and multiple identical microarrays were printed in a 96-well plate-like format [97,98]. They also synthesized and purified 217 fluorescently labeled peptides derived from the C-terminal residues of mouse proteins. All possible interactions of these 157 PDZ domains with the 217 genome-encoded peptides were then examined by the fluorescence polarization assay [97]. The PDZ domains microarrays identified interactions of moderate to high affinity (*K*_D _≤ 10 μM) in a high-throughput format, with a moderate false-positive rate of 19% and an even lower false-negative rate of 14% [98]. The results were subsequently used to build a model with a position-specific scoring matrix (PSSM) that predicts the selectivity of the PDZ domain [97,99]. Using this model, MacBeath and coworkers screened 31,302 peptide sequences corresponding to the C-termini of all translated open reading frames in the mouse genome and found no less than 18,149 PDZ-peptide interactions. This suggests that obtaining comprehensive information on PDZ-peptide interactions may be very helpful in supporting future biological investigations of target protein functions [97,99].

#### Peptide library approaches: Phage display and SPOT synthesis

Because PDZ domains recognize only short linear motifs in their target proteins, peptide library approaches are being used to define the binding specificity of PDZ domains, to confirm known PDZ interactions, to optimize the PDZ-binding ligands, and to find putative PDZ-binding partners [31-44,100].

**Phage display **is a high-throughput approach in which libraries of more than 10^11 ^random peptides or proteins are expressed on the surfaces of phage particles, which harbor short randomised DNA stretches that encode for the oligopeptide to be displayed for studying PDZ-ligand interactions [32]. After typically several rounds of 'panning' the binding peptide candidates are identified by isolating single phages and sequencing their DNA [101]. Given that most PDZ domains recognize the free C-termini tail of target proteins, C-terminally displayed peptides have been developed [31,32,39,40,73,102]. Songyang *et al*. (1997) examined peptide-binding specificities of 9 PDZ domains by using the oriented peptide library to elucidate relative preferences for certain amino acids at a given position of PDZ-binding ligands [31]. Kurakin *et al*. (2002) developed the target-assisted iterative screening (TAIS) method, a simple and rapid 2-step procedure for *in vitro *affinity selection of specific binding partners from molecules with enormous molecular diversities to the target molecule of interest [34]. This method has been applied to a commercial phage-displayed cDNA library with a PDZ domain as a target to investigate the selectivity and promiscuity of the interactions [39]. Tonikian *et al*. (2008) used C-terminal phage-displayed random peptide libraries containing greater than 10 billion random peptides to analyze the binding specificities of 145 PDZ domains (from 57 *C. elegans *and 88 human proteins) [73].

**SPOT synthesis **allows the parallel synthesis and screening of thousands of cellulose membrane-bound peptides, and has been applied to study PDZ-mediated interactions [44,103]. For example, Wiedemann *et al*. (2004) generated a peptide library comprising 6223 C-termini of human proteins by SPOT synthesis of inverted peptides to obtain an overview of the space of target sequences for 3 PDZ domains from AF6, ERBIN, and SNA1 proteins, respectively [103]. On the basis of the ligand preferences detected for these PDZ domains, they designed focused peptide libraries (profile libraries) and quantified the binding affinity contributions of the 4 C-terminal ligand residues. The authors studied the binding specificities of PDZ domains and established the relationship between the C-terminal ligand sequences and the corresponding K_D _values. Finally, they predicted putative PDZ-binding partners on the basis of the SWISS-PROT database.

### Classification of PDZ domains

Recent studies on proteomic applications that use protein arrays and peptide libraries have generated a wealth of information on protein-protein (or peptide) interactions (PPIs) [71,72,93-95]. In addition, the existing classification of PDZ domains - (1) class I domains, which recognize the motif S/T-X-Φ; (2) class II domains, which recognize the motif Φ-X-Φ; and (3) class III domains, which recognize the motif D/E-X-Φ as their preferred C-terminal motif, where Φ represents a hydrophobic residue - has been challenged because of the importance of other upstream positions within the PDZ ligand, such as -3 or -4 position (position 0 referring to the C-terminal residue), to the binding specificity of target proteins [36,42,71,73,74,104]. By analyzing a total of 72 PDZ domains corresponding to 2,998 ligands, Tonikian *et al*. (2008) suggested the 16 classes of PDZ domains, which are defined by the following C-terminal motifs: **1a **(ϕ-[K/R]-X-S-D-V); **1b **(Ω-[R/K]-E-T-[S/T/R/K]-ϕ); **1c **(ϕ-ϕ-E-T-X-L); **1d **(E-T-X-V); **1e **(T-W-ψ); **1f **(Ω-Ω-T-W-ψ); **1g **(ϕ-ϕ-ϕ-[T/S]-[T/S]-Ω-ψ]; **2a **(F-D-Ω-Ω-C); **2b**(W-X-Ω-D-ψ); **2c **(W-Ω-ϕ-D-ψ); **2d **(ϕ-ϕ-X-[E/D]-ϕ-ϕ-ϕ); **2e **(ϕ-ϕ-ϕ-ϕ); **2f **([D/E]-ϕ-Ω-ϕ); **3a **(Wx[S/T]-D-W-ψ); **4a **(Ω-ϕ-G-W-F); ϕ, hydrophobic (V, I, L, F, W, Y, M); Ω, aromatic (F, W, Y); ψ, aliphatic (V, I, L, and M); and X, nonspecific [73]. They also suggested that their specific map for the PDZ domain family will be able to predict natural protein interactions [73]. Further studies with independent methods would be necessary to verify their classification, because the 72 PDZ domains investigated by Tonikian *et al*. (2008) may not be enough to represent the whole PDZ 'domainome'.

### Bioinformatics and other methods for finding putative PDZ-binding partners

Studies with PDZ microarrays and peptide libraries have focused on generating information on PDZ-mediated interactions, generating a key resource to investigate biological networks and signaling pathways within cells [28-44,97-99]. This information needs to be comprehensively deposited in publicly available repositories, such as iSPOT, DOMINO, and PDZBase [105-108], in order to maximally accelerate the discovery of novel PDZ-mediated interactions in cells. PDZBase is a unique database that contains information extracted from the literature of all known PDZ domain-mediated protein-protein interactions obtained from *in vivo *(coimmunoprecipitation) or *in vitro *experiments (GST-fusion or related pull-down experiments) [108].

However, the information on interactions derived from high-throughput methods should be interpreted with caution and verified by other independent methods, such as Y2H and co-IP. For example, MacBeath and coworkers predicted 18,149 PDZ-peptide interactions from PDZ microarrays [97,98]. Among them, 710 proteins were proposed to be binding partners for the Dvl PDZ domain [97]. Because Dvl proteins (Dvl1, Dvl2, and Dvl3 in mammals) play diverse roles in Wnt signaling [109], identification of their binding partners is key to understanding their biological functions. Although we cannot exclude the possibility that unexpected Dvl-binding proteins may exist in the predicted data [97], no known Dvl PDZ-binding partners such as Dapper [65] and Daple [110] have been found in this data set, highlighting the importance of further verification of the proposed binding partners by other methods. Furthermore, there is a discrepancy between the list of predicted Dvl PDZ binding partners reported by Tonikian *et al*. (2008) who were using phage-displayed oriented peptide libraries and that by Stiffler *et al*. (2007) resulting from the use of PDZ domain microarrays [97]. Ten potential binding partners of the Dvl2 PDZ domain predicted by Tonikian *et al*. are not found in the prediction list of Stiffler *et al*. In addition, because neither study considered the expression profiles and subcellular localizations of the proposed PDZ-binding partners, the number of real binding candidates for a specific protein is expected to be significantly lower than that reported. siRNA experiments are needed to verify putative interactions *in vivo*. Along these lines, Cui *et al*. (2007) conducted a proteomic analysis of the interactions of neuronal signaling proteins with human PDZ domains (>6,500 interactions) using an ELISA-based assay [94]. They found that the Tat-NR2B9c peptide, which is a Tat peptide consisting of the nine COOH terminal residues of the NR2B subunit, binds specifically to PSD-95 family members (PSD-95, PSD-93, SAP97 and SAP102) and Tax interaction protein 1 (TIP1). As they suppressed the Tat-NR2B9c-binding proteins in primary murine neuron culture by RNA interference, remarkably, neurons lacking PSD-95 or nNOS, but no other PDZ domains, exhibited reduced excitotoxic vulnerability [94].

Taken together, optimal use of all the databases compiling the interactions obtained by different methods will reduce the time and expense of finding a specific PDZ-binding partner for further studies at a genome-wide level, and will also aid its functional characterization [24,73,111].

## Regulation of PDZ-mediated interactions

As PDZ domains interact with many proteins, understanding the regulatory mechanisms of PDZ-mediated interactions is important to gain insight into biological processes. Posttranslational modification, autoinhibition, and allosteric interaction have been proposed to regulate PDZ-mediated interactions.

### Phosphorylation within the PDZ ligand modulates PDZ protein-protein interactions

**Figure 5:**
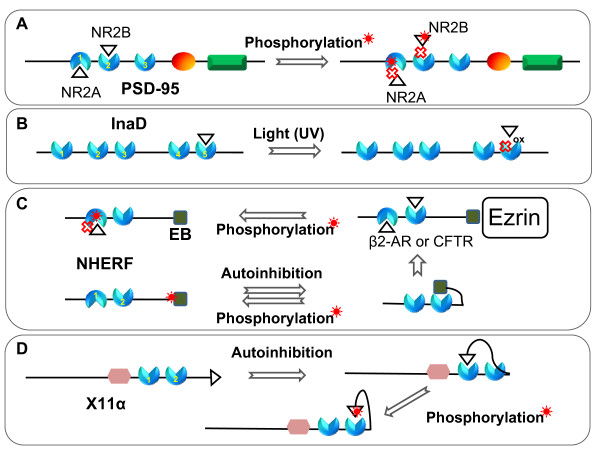
**Posttranslational modifications on the PDZ ligands or PDZ domains modulate PDZ protein-protein interactions**. (A) Phosphorylation of the PDZ ligand or PDZ domain inhibits PDZ interactions. The cross represents a reduced or abolished interaction. (B) Formation of intramolecular disulfide bond (symbol, 'ox') in the PDZ domain prevents binding of the other binding partner. (C, D) Phosphorylation or competitive binding changes the autoinhibited conformation.

Phosphorylation of Ser, Thr, or Tyr within the PDZ ligand can modulate PDZ-mediated interactions (Table 1 and Figure 5). For example, the interaction between the NR2B subunit of the NMDA receptor with PSD-95 is negatively modulated by phosphorylation (Figure 5A). The PDZ ligand (LSSIE**S**DV_COOH_) of NR2B at the p(-2) site is phosphorylated by CK2 *in vivo *(although S(-2) does not match the substrate consensus motif of CK2), which disrupts its interaction with PSD-95 and decreases the surface expression of NR2B in neurons [112]. The authors also reported that CK2 colocalizes with NMDAR in dendrites and at some excitatory synapses [112,113]. Kim and coworkers showed that phosphorylation by PKA at the p(-2) site within the PDZ ligand (ANRRT**T**PV_COOH_) of stargazin, which is a transmembrane AMPA receptor regulatory protein, abrogates its binding to PSD-95 PDZ1 domains and thereby regulates synaptic AMPAR function [114,115]. The disruption of PDZ-mediated interactions by phosphorylation can be rationalized by the elimination of a possible hydrogen bond donor sidechain; the side chain of an unmodified Ser or Thr residue at the p(-2) can form a hydrogen bond with the N-3 nitrogen of the His residue at position αB-1 for the PDZ class I domain, and phosphorylation of the PDZ ligand in this position destroys this possibility, resulting a in loss of PDZ-based interactions [78].

**Table 1:** Phosphorylation of the PDZ ligand by serine or threonine kinase modulates PDZ protein-protein interactions

PDZ-containing proteins	Binding partner(s)	PDZ ligand^a^	Kinase(s)	Ref.
PSD-95	Kir2.3	ISYRREE**S**RI	PKA (RR/KX**S/T**)^b^	Cohen *et al*. [187]

PSD-95	Stargazin	ANRRT**T**PV	PKA (RR/KX**S/T**)^b^	Choi *et al*. [115] Chetkovich *et al*. [114]

PSD-95/SAP102	NR2B subunit of NMDAR	LSSIE**S**DV	CK2 (**S/T**XXE/D)^b^	Chung *et al*. [112]

GRIP1	GluR2 subunit of AMPAR	YNYGIE**S**VKI	PKC (RXX**S/T**XR/K)^b^	Matsuda *et al*. [116] Chung *et al*. [117]

PSD-95	NR2C subunit of NMDAR	RRVS**S**LESEV	PKA/PKC	Chen *et al*. [127]

PSD-95/SAP97	LRP4	ERKL**S**SESQV	CaMKII (RXX**S/T**) ^b^	Tian *et al*. [119]

^a ^Phosphorylation sites of PDZ ligands are underlined and in bold.

^b ^Kinase consensus phosphorylation motif (where X indicates any amino acid) [188,189].

Besides phosphorylation of the p(-2) site within the PDZ ligand, other positions in the ligand can also be phosphorylated [55,86,116,117]. The Ser residue at p(-4) site in the C-terminal PDZ ligand (YNYGIE**S**VKI_COOH_) of the AMPA receptor GluR2 subunit is phosphorylated by PKC *in vitro *and *in vivo *[116,117]. Coimmunoprecipitation and *in vivo *binding studies have shown that phosphorylation significantly decreases binding of GluR2 to the PDZ domain of GRIP1/2 but not of PICK1. Lin and Huganir reported that phosphorylation of GluR2 and binding to PICK1 dynamically regulate GluR2 recycling [118]. Tian *et al*. (2006) showed that CaMKII phosphorylates the C-terminal cytoplasmic region of LRP4 at Ser-1900, p(-5) site, of the C-terminal tail (ERKL**S**SESQV_COOH_), which suppresses the interaction of the protein with PSD-95 and SAP97 [119]. The reason for the decrease in PDZ binding affinity by phosphorylation at the -4 and -5 positions of residues in the PDZ ligand remains unclear. Zhang and coworkers have shown by structural and biochemical studies that domain-swapped dimerization of the ZO-1 PDZ2 domain plays a crucial role in the interaction with the C-terminus of the connexin43 protein (referred to as Cx43 peptide, ASSRPRPDDLEI) [55]; this interaction is regulated by phosphorylation of Ser residues at the -9 and -10 positions in the PDZ ligand of Cx43. These Ser residues are substrates for the kinases Akt and PKC [120-125]. NMR studies suggest that the phosphorylation of the Ser residues at p(-9) and p(-10) sites may interfere with the charge-charge interaction network formed by Cx43 and the residues at the dimer interface of ZO-1 PDZ2 [55].

To examine the effect of ligand position-dependent phosphorylation of the PDZ ligand, Volkmer and coworkers developed a modified SPOT synthesis technique that generated 3 arrays, each containing the 100 PDZ-binding sequences and also all possible phosphorylated variants for the 3 PDZ domains from AF-6, ERBIN, and SNA-1 proteins [38]. The interactions of 344 peptides for AF-6 PDZ, 319 peptides for ERBIN PDZ, and 355 peptides for the SNA-1 (α-1-syntrophin) PDZ domains showed that phosphorylation of the PDZ ligand at p(-2) (<50% residual binding activity [rba]) and at p(-1) (~50% rba) significantly inhibited PDZ-mediated interactions; phosphorylation at p(-4), (-7), and (-8) only slightly affected the interactions (~80% rba), depending on the PDZ domain; and phosphorylation at p(-3), (-5), (-6), (-9), or (-10) had little or no influence on the interactions (>80% rba). Although the PDZ domain of AF-6 is recognized as a class II PDZ domain, phosphorylation at p(-2) site disrupts the interaction between AF-6 PDZ and the C-terminal ligand (S**T**EV) of BCR (~30% rba). Data on the phosphorylation sites of PDZ ligands and the roles of phosphorylations of the PDZ ligands will be useful to elucidate the regulatory mechanism of PDZ-mediated interactions, even if the kinases that phosphorylate the PDZ ligands remain unknown.

While many studies have reported that phosphorylation at the C-terminus of proteins negatively modulates PDZ interactions, others have shown that phosphorylation can also promote PDZ interactions [86,126]. Interestingly, a study by Roche and coworkers documented that phosphorylation of a PDZ-binding motif did not affect PDZ interactions: phosphorylation by PKA or PKC of the p(-6) site within the C-terminus of the NR2C subunit of NMDAR did not change the binding of the PSD-95 PDZ3 or the surface expression of NR1/NR2C NMDA receptors [127]. Surprisingly, a phosphomimetic mutation accelerated channel kinetics, suggesting that phosphorylation alters the function of NMDA receptor channels. These results suggest that phosphorylation of the PDZ binding motif of a target protein might not affect association with the PDZ domain but can still play a role in the functioning of its target protein.

### Disulfide bond formation blocks PDZ protein-protein interactions

Although phosphorylation is important in regulating PDZ protein-protein interactions, intramolecular disulfide bond formation in PDZ domains can also modulate binding [128,129]. For example, the PDZ5 domain of InaD, a multiple PDZ domain-containing protein in photoreceptor cells of the fruit fly (Figure 1), exists in a redox-dependent equilibrium between 2 conformations: the reduced form, which is similar to the structure of other PDZ domains, and the oxidized form, in which the ligand binding site is distorted through formation of a strong intramolecular disulfide bond between 2 cysteines situated in the βC strand and the αB helix (Figure 5B) [128,129]. This provides the first evidence that disulfide bond formation is able to change the conformation of the PDZ domain and to regulate its function.

### Phosphorylation on the PDZ domain itself negatively modulates PDZ interactions

Several studies have reported that phosphorylation on the PDZ domain itself may also disrupt PDZ protein-protein interactions (Figure 5A and 5C). For example, Luca and coworkers found that activation of the NMDA receptor induces a CaMKII-dependent phosphorylation of SAP-97 or PSD-95 [130]. The protein SAP-97 is directly associated with NR2A protein through its PDZ1 domain, and phosphorylation of Ser-232 in SAP-97 by CaMKII disrupts NR2A interaction both *in vitro *and *in vivo*. The authors also identified a CaMKII-dependent phosphorylation on the PDZ domain of PSD-95 [130]. CaMKII phosphorylation of Ser-73 of PSD-95 causes NR2A dissociation from PSD-95, but does not interfere with the binding of NR2B to PSD-95 [130]. This phosphorylation of PSD-95 negatively regulates spine growth and synaptic plasticity [131]. Remarkably, Ser-232 in the PDZ1 domain of SAP-97 and Ser-73 in PDZ1 domain of PSD-95 are located in the αB-helix structure, which is the binding site of the PDZ domain. These results suggest that phosphorylation at a Ser or Thr residue of the binding sites of PDZ domains plays an important role in regulating PDZ-mediated interactions.

Another example is the phosphorylation site (Ser-77) of the first PDZ domain (PDZ1) of NHERF-1, a signaling adaptor protein containing 2 PDZ domains at the N-terminus and an ezrin-radixin-moesin (ERM) domain-binding (EB) region at the C-terminus [132,133]. The phosphorylation of Ser-77, located on the αB-helix on the PDZ domain, by protein kinase C (PKC) attenuates its binding to physiological targets such as the β_2_-adrenergic receptor and sodium-phosphate cotransporter type IIa (Figure 5C) [133,134]. The phosphorylation at Ser-162 of the second PDZ (PDZ2) domain in NHERF-1 has also been reported [135]. Raghuram *et al*. (2003) showed that this phosphorylation lowers the PDZs affinity for the CFTR C terminus and disrupts the bivalent PDZ domain interaction of NHERF-1 (Figure 5C) [135].

### Autoinhibited conformation of PDZ-containing proteins

Some PDZ-containing proteins have a PDZ-binding motif at their C-terminal tail. The binding site of the PDZ domain in these proteins can be occupied by their own C-terminal sequences, thereby inhibiting the binding of the PDZ ligand [45,61,136-141]. Here, we discuss three PDZ-containing proteins that adopt the auto-inhibitory conformation.

#### NHERF-1

Two independent groups have reported that the C-terminal tail of the NHERF-1 protein binds to its own PDZ2 domain (Figure 5C) [45,136]. Data from solution SAXS show that the C-terminal EB region in NHERF-1 folds back to PDZ2 [136], which is supported by recent NMR and circular dichroism (CD) studies showing the presence of specific intramolecular interactions between PDZ2 and the C-terminal EB regions [139]. Remarkably, the last residues in the C-terminus of NHERF-1 adopt an α-helix conformation when bound to PDZ2, which is comparable to an extended conformation of a typically bound PDZ ligand [138,139] and this α-helical conformation of the EB region is accommodated in the peptide binding pocket of PDZ2. GST pull-down experiments and surface plasmon resonance (SPR) experiments indicate that the intramolecular interactions between PDZ2 and the EB region compete with the binding of extrinsic ligands [45,139]. In addition, studies on the effect of phosphorylation on the autoinhibitory conformation of NHERF-1 by solution SAXS and binding assays suggest that this conformation could be disrupted by protein kinase C (PKC) phosphorylation at Ser-339 and Ser-340 in the C-terminal domain of NHERF-1. In line with this, the PKC phosphorylation-mimicking mutant NHERF(S339D/S340D) displays a higher binding affinity for its extrinsic ligand C-CFTR than wild-type NHERF-1 does [136]. The autoinhibited conformation of NHERF-1 can also be regulated by the binding of ezrin (Figure 5C). Li *et al*. (2009) used small-angle neutron scattering (SANS) to demonstrate that the C-terminal EB region binds to ezrin, which induces conformational changes in two region of NHERF-1: the region linking PDZ2 and the C-terminal EB region and also that linking the PDZ1 and PDZ2 domains [140]. The authors suggest that this long-range interdomain conformation in NHERF1 bound to ezrin increases the binding capabilities of both PDZ domains [140].

The **X11α **protein, involved in regulating neuronal signaling, trafficking and plasticity[142], contains a central PTB domain and 2 C-terminal PDZ domains (PDZ1 and PDZ2 arranged in tandem). Zhang and coworkers found that the C-terminal tail of X11α folds back and binds to the first PDZ domain, suggesting that the binding site of PDZ1 is closed (Figure 5D) [61]. The authors hypothesize that phosphorylation on the C-terminal tail of X11α might re-open the binding site of the PDZ1 domain. To test this hypothesis, they made a phosphorylation mimic peptide wherein the highly conserved Tyr(-1) residue was substituted with the Glu(-1) residue at the C-terminal tail of X11α. Interestingly, this mutant peptide did not bind to the PDZ1 domain but did bind to the PDZ2 domain of X11α, suggesting that phosphorylation might lead to conformational changes in the autoinhibited PDZ-containing protein and also changes in the binding selectivity of PDZ domains in X11α. However, the tyrosine kinase driving this phosphorylation remains to be determined (Figure 5D).

**Tamalin**, which is also called the GRP1-associated protein [143], contains an N-terminal alanine-rich region, a central PDZ domain, and a C-terminal Leu-zipper domain (Figure 1) [144-146]. Sugi *et al*. (2007) have reported the crystal structure of the autoinhibitory PDZ domain of tamalin [141]. In the absence of mGluR protein, tamalin self-assembles into an autoinhibited conformation through its PDZ domain and its C-terminal PDZ ligand. The C-terminus of mGluR protein can competitively bind to the PDZ domain of tamalin at a high concentration, thereby disrupting weak inhibitory interactions, suggesting that the PDZ domain of tamalin switches between the trafficking-inhibited and -active forms, depending on the association with mGluR [141].

### Allosteric regulation of PDZ-mediated protein interactions

Recent studies provide evidence that protein-protein interactions influence the changes in the time scale and amplitude of protein motion within a domain as well as long-range coupled motions between protein domains [20,40,140,147-149]. Thus, several studies have examined the effect of allostery in PDZ-containing proteins [20,40,140,147-150], and some have shown that allosteric interactions modulate the binding preferences of PDZ domains [20,40]. Van den Berk *et al*. (2007) investigated the binding preferences of the 5 PDZ domains in protein tyrosine phosphatase PTP-BL by using a random C-terminal peptide lambda phage display library [40]. They found that the potential of PDZ2 to interact with class III-type ligands can be modulated by the presence of PDZ1. Structural studies have shown that the interaction of PDZ1 with the surface area of PDZ2 opposite the binding groove changes the binding specificity of PDZ2.

Furthermore, Li *et al*. (2009) reported that the binding of ezrin to NHERF1 increases the binding capabilities of both PDZ domains (Figure 5C) [140]. They further demonstrated that NHERF1 undergoes significant conformational changes in the regions linking PDZ1 and PDZ2 and also those linking PDZ2 and the C-terminal ezrin-binding domain when it forms a complex with ezrin. Together, these results imply that the allosteric behavior in PDZ-mediated protein-protein interactions plays an important role in regulating these interactions.

### Deregulation of PDZ-mediated interactions

Consistent with the observations that PDZ protein-protein interactions regulate diverse biological functions, deregulation of PDZ interactions has been linked to various diseases such as cancer [25,44,48,151-155]. Therefore, small molecules, peptides, and peptidomimetics that regulate specific PDZ-mediated interactions have attracted significant attention because of their potential to elicit therapeutic benefits [47,149,153,155-162]. Since these compounds that target specific PDZ-mediated interactions also have a wide-ranging potential as tools for elucidating disease pathways, extensive studies are in progress to discover more potent compounds that inhibit PDZ-mediated interactions [47,152,156-165].

## Conclusions

Experimental and theoretical studies have been extensively conducted to understand PDZ-mediated interactions. Many studies, however, have used a single PDZ domain despite the presence of multiple copies of the PDZ domain or combination of other interaction modules in proteins. Accumulating studies show, however, that the binding preferences of tandem arrangements within proteins with multiple PDZ domain differ from those of proteins with a single PDZ domain [40,63,166-168], implying that careful examination of the binding properties of proteins containing tandem PDZ domains or PDZ domains combined with other interaction module is required. In addition, the biological significance and mechanistic details of multiple PDZ domain-containing proteins still remain to be investigated.

Because PDZ-containing proteins may interact with dozens of proteins, it is paramount to understand the regulatory mechanisms of PDZ protein-protein interactions such as phosphorylation, disulfide bond formation, autoinhibition, competitive binding, and allostery. Phosphorylation of PDZ ligands is likely to be a major regulatory mechanism, but the kinases catalyzing these phosphorylations are often yet to be characterized. We expect that proteomics and bioinformatics can help to determine these kinases and also the phosphorylation sites of the proteins of interest [169-173]. Since other posttranslational modification of proteins such as acetylation have also been proposed [174], future studies also need to focus on identifying and characterizing such unrecognized modifications of PDZ-mediated interactions [175,176]. An alternative regulatory mechanism that has been proposed for the formation and stabilization of protein complexes is the binding of many PDZ domains to phosphoinositide (PtdInsP)-containing lipid membranes [177-183]. A complete understanding of the regulatory mechanisms of PDZ-mediated interactions will enhance our knowledge of many cellular and biological processes.

## List of abbreviations

AR: Alanine Rich region; LZ: Leucine-Zipper; GPCR: G Protein Coupled Receptor; NHERF-1: Na+/H+ Exchanger Regulatory Factor; β2-AR: β_2_-adrenergic receptor; GRK-5: G-protein-coupled receptor kinase; DIX: Dishevelled and Axin; DEP: Dishevelled, Egl-10, and Pleckstrin; PKA: Protein Kinase A; NMDAR: N-Methyl-D-Aspartate Receptors; NorpA: No-receptor Potential A; CK2: Casein Kinase II; PKC: Protein Kinase C; GRIP: Glutamate receptor interaction protein; NMDAR: PKA: Protein Kinase A; LRP4: Low-density lipoprotein Receptor (LDLR)-related Protein 4; CaMKII: Ca2+/calmodulin-dependent protein kinase II; HtrA: High-Temperature Requirement A; SH3: Src homology 3; GK: guanylate kinase-like; Y2H: yeast two-hybrid.

## Competing interests

The authors declare that they have no competing interests.

## Authors' contributions

Both authors wrote the manuscript and approved its final version.
